# Interleukin-9 protects from microglia- and TNF-mediated synaptotoxicity in experimental multiple sclerosis

**DOI:** 10.1186/s12974-024-03120-9

**Published:** 2024-05-14

**Authors:** Livia Guadalupi, Valentina Vanni, Sara Balletta, Silvia Caioli, Francesca De Vito, Diego Fresegna, Krizia Sanna, Monica Nencini, Gloria Donninelli, Elisabetta Volpe, Fabrizio Mariani, Luca Battistini, Mario Stampanoni Bassi, Luana Gilio, Antonio Bruno, Ettore Dolcetti, Fabio Buttari, Georgia Mandolesi, Diego Centonze, Alessandra Musella

**Affiliations:** 1 Department of Systems Medicine, University of Rome Tor Vergata, Rome, 00133 Italy; 2 Synaptic Immunopathology Lab, IRCCS San Raffaele Roma, Rome, 00166 Italy; 3 Unit of Neurology, IRCCS Neuromed, Pozzilli (Is), 86077 Italy; 4 Molecular Neuroimmunology Unit, IRCCS Fondazione Santa Lucia, Via del Fosso di Fiorano 64, Rome, 00143 Italy; 5 Neuroimmunology Unit, IRCCS Fondazione Santa Lucia, Via del Fosso di Fiorano 64, Rome, 00143 Italy; 6 Ph.D. Program in Neuroscience, Department of Systems Medicine, University of Rome Tor Vergata, Rome, 00133 Italy; 7 Department of Human Sciences and Quality of Life Promotion, University of Rome San Raffaele, Rome, 00166 Italy

**Keywords:** Experimental multiple sclerosis, Neuroinflammation, Synaptopathy, Microglia activation, Inflammatory cytokines, Glutamate transmission

## Abstract

**Background:**

Multiple sclerosis (MS) is a progressive neurodegenerative disease of the central nervous system characterized by inflammation-driven synaptic abnormalities. Interleukin-9 (IL-9) is emerging as a pleiotropic cytokine involved in MS pathophysiology.

**Methods:**

Through biochemical, immunohistochemical, and electrophysiological experiments, we investigated the effects of both peripheral and central administration of IL-9 on C57/BL6 female mice with experimental autoimmune encephalomyelitis (EAE), a model of MS.

**Results:**

We demonstrated that both systemic and local administration of IL-9 significantly improved clinical disability, reduced neuroinflammation, and mitigated synaptic damage in EAE. The results unveil an unrecognized central effect of IL-9 against microglia- and TNF-mediated neuronal excitotoxicity. Two main mechanisms emerged: first, IL-9 modulated microglial inflammatory activity by enhancing the expression of the triggering receptor expressed on myeloid cells-2 (TREM2) and reducing TNF release. Second, IL-9 suppressed neuronal TNF signaling, thereby blocking its synaptotoxic effects.

**Conclusions:**

The data presented in this work highlight IL-9 as a critical neuroprotective molecule capable of interfering with inflammatory synaptopathy in EAE. These findings open new avenues for treatments targeting the neurodegenerative damage associated with MS, as well as other inflammatory and neurodegenerative disorders of the central nervous system.

**Supplementary Information:**

The online version contains supplementary material available at 10.1186/s12974-024-03120-9.

## Introduction

Multiple sclerosis (MS) is an inflammatory and neurodegenerative autoimmune disease resulting in a diffuse demyelination, neuroinflammation, neuroaxonal loss and dysfunction of the central nervous system (CNS) [1, 2]. In the last decade, both clinical and preclinical studies in MS and in its animal model, the experimental autoimmune encephalomyelitis (EAE), have highlighted degenerative changes of the grey 4matter as crucial pathological events independent of demyelination [3, 4].One of the most prominent features of this neuronal damage is a synaptic dysfunction and loss, named synaptopathy, characterized by a marked imbalance between glutamatergic and GABAergic transmissions, caused by the neuroinflammatory milieu that characterizes MS and EAE brains [3, 5, 6]. Microglia and astrocytes extensively contribute to MS neuroinflammation and greatly modulate synaptic transmission by releasing proinflammatory cytokines. In particular, in the striatum of EAE mice, the proinflammatory cytokine TNF, mainly released by activated microglia and macrophages, enhance excitatory transmission acting at postsynaptic glutamate receptor sites [7–9]. The long-lasting perturbation of synaptic homeostasis ultimately results in excitotoxic neurodegeneration. Since primary alterations of the synaptic compartments are potentially reversible, synaptopathy represents an attractive therapeutic target in MS [10].

Interleukin-9 (IL-9) is a pleiotropic cytokine which affects both immune and non-immune cells, acting on both peripheral and CNS compartments [12]. IL-9 has emerged as an important signature within the cytokine microenvironment that characterize MS pathophysiology [11]. Accordingly, levels of IL-9 in the cerebrospinal fluid (CSF) of relapsing-remitting (RR) MS patients were shown to inversely correlate with indexes of inflammation, neurodegeneration, disability progression, and response to first line therapies [12]. Furthermore, IL-9 was shown to be expressed in the CNS of progressive MS patients and its expression was mainly associated with macrophage and CD4^+^ T cell infiltration and microglial activation [13].

A large number of different cell types express IL-9R, such as CD4^+^ and CD8^+^ T cells, B cells, macrophages, mast cells, and eosinophils [11].Importantly, IL-9 was shown to regulate and decrease the activation phenotype of human macrophages [13] and to inhibit TNF release from LPS stimulated- human monocytes [14]. Moreover, a growing body of evidence has shown the expression of IL9R in CNS-resident cells, including microglia cells [11, 15, 16].

Despite the encouraging data related to IL-9 in MS patients, studies in animal models of MS aimed to better characterize its role during the pathology, did not reach conclusive results. Indeed, studies performed using systemic administration of recombinant IL-9, IL-9 blockers, or IL-9 receptor (IL-9R) knockout mice have yielded conflicting results [17–20]. Moreover, stimulation of IL-9 production from T cells has been shown to either ameliorate or worsen EAE disease in mice, depending on whether treatment occurred before or concurrently with EAE induction, respectively [21]. The mRNA and protein level of IL-9 increase in EAE spinal cords, and such increase correlates with a worse clinical score [22]. Finally, IL-9R expression was shown to be upregulated in the brain and spinal cord during EAE development [15].

Building upon promising findings concerning the role of IL-9 in MS, this study aimed to elucidate its controversial role in the mouse model of the disease and shed light on its effects during neuroinflammatory conditions. To achieve this goal, we conducted three different pharmacological treatments with IL-9. Through a combination of clinical score analysis, electrophysiological recordings, immunofluorescence, and biochemical studies, we uncovered the peculiar neuroprotective properties of IL-9, demonstrating its efficacy in counteracting inflammatory synaptopathy and clinical deficits during EAE.

## Materials and methods

### Mice

Animals employed in this study were 6–8-week-old C57BL/6 female mice, obtained from Charles-River (Milan, Italy). Mice were housed under constant conditions in an animal facility with a regular 12 h light/dark cycle. Food and water were supplied ad libitum. All the efforts were made to minimize the number of animals used and their suffering. In particular, when animals experienced hindlimb weakness, moistened food and water were made easily accessible to the animals on the cage floor. Mice with hindlimb paresis received glucose solution by subcutaneous injection or food by gavage during the entire procedure. In the rare presence of a tetraparalyzed animal, mice were sacrificed. Minipump-implanted mice were housed in individual cages endowed with special bedding (TEK-Fresch, Envigo, Casatenovo (LC), Italy) in order to avoid skin infections around the surgical scar. Animal experiments were performed according to the Internal Institutional Review Committee, the European Directive 2010/63/EU and the European Recommendations 526/2007, and the Italian D.Lgs 26/2014.

### EAE model

Chronic-progressive EAE was induced as previously described [23]. Six-eight weeks old C57BL/6 female mice were active immunized with an emulsion of mouse myelin oligodendrocyte glycoprotein peptide 35–55 (MOG35–55, 85% purity; 0,66 mg/ml; Espikem, Prato, Italy) in Complete Freund’s Adjuvant (CFA; Difco, Los Altos, CA, USA), followed by intravenous administration of pertussis toxin (500 ng; Merck, Milan, Italy) on the day of immunization and two days post immunization (dpi). Control mice, hereafter referred to as CFA, received the same treatment as EAE mice without the MOG peptide, including complete CFA and Pertussis toxin. Animals were daily scored for clinical symptoms of EAE according to the following scale: 0 no clinical signs; 1 flaccid tail; 2 hindlimb weakness; 3 hindlimb paresis; 4 tetraparalysis; and 5 death due to EAE; intermediate clinical signs were scored by adding 0.5. The body weight (g) of each mouse was measured over the course of the disease course and compared with body weight value at 0 dpi.

### Grip strength test

All mice were tested for grip strength performance using the Grip Strength Meter (Ugo Basile, Italy), as in [24] at 2,7,14 and 21 dpi. The Grip Strength Meter consisted of a steel wire grid (8 × 8 cm) connected to an isometric force transducer. Mice were lifted by their tail so that they grasp the grid with their paws. Mice from the two experimental groups were then gently pulled backward until they released the grid and the maximal force in newtons (N) exerted by the mouse before losing the grip was measured. The mean of three consecutive measurements for each animal was calculated and values were normalized by mouse body weight.

### BV2 immortalized murine microglial cell line

The BV2 immortalized murine microglial cell line was constructed by infecting primary microglia with a v-raf/v-myc oncogene-carrying retrovirus (J2). The murine BV2 microglia were cultured in DMEM supplemented with 10% FBS, 100 U/ml penicillin and 100 µg/ml streptomycin, and were maintained in a humidified incubator with 5% CO2. BV2 cells were in vitro activated for 6 h, 18 h and 24 h with a Mix of Th1-specific proinflammatory cytokines: 100 U/mL IL-1β (Euroclone, Milan, Italy), 200 U/mL tumor necrosis factor (TNF, Miltenyi Biotec, Bologna, Italy), and 500 U/mL interferon γ (IFNγ, Becton Dickinson, Milan, Italy). Only the 24 h activated BV2 cells were afterwards treated with IL-9 (100 μm) for 6 h. Then, 5 × 10^5 to 1 × 10^6 cells were put on single striatal slices (for 60 min) and whole-cell patch-clamp recordings were made as above. For biochemical evaluation, following IL-9 treatment, activated cells were cultured for 3 h in a non-activated medium. Cell medium was used for ELISA experiment (see after).

### Western blot

Whole striata dissected from EAE mice (i.p. treatment starting from 0 dpi) in the acute phase of the disease (24 dpi) were homogenized in RIPA buffer plus protease inhibitor mixture (Sigma) and sonicated. After sonication, the homogenates were centrifuged at 13,000 ×g for 20 min and the supernatant was collected. Protein content was quantified according to the Bradford Assay method (Thermofisher). 20 µg of striatal extract were loaded onto a sodium dodecyl-sulfate polyacrylamide gel. Gel was blotted onto Nitrocellulose membrane (Millipore). WB experiments were performed as previously described [24] and the following primary antibodies were used: o/n mouse anti-TREM2 (1:1000,R&D system); mouse anti- β-actin (1:20000, Sigma) for 1 h room temperature (RT). Membranes were incubated with a secondary HRP-conjugated IgG anti-mouse (1:10000 for β-actin and 1:2000 for TREM2, 1 h RT, Abcam). Results were presented as data normalized to β-actin and EAE-VHL or CTRL values. *N* = 4–5 per group for EAE mice.

### TNF ELISA measurement

Conditioned medium from BV2 cells non-activated (control), Th1 activated and Th1 activated in the presence of IL-9 (see above) was harvested and centrifuged at 1,200 rpm in microfuge in order to remove any dead or detached cells. TNF ELISA was performed according to manifacturer’s guide (Biotechne). The medium (*n* = 4–5 samples for each condition) were run in the same assay. Quantification of cytokine content was made according to standard curve in the linear range (from 10 to 700 pg/ml).

### Electrophysiology

Mice were killed by cervical dislocation, and corticostriatal coronal slices (200 μm) were prepared from fresh tissue blocks of the brain with the use of a vibratome. A single slice was then transferred to a recording chamber and submerged in a continuously flowing ACSF (34 °C, 2–3 mL/min) gassed with 95% O2–5% CO2. The composition of the control ACSF was (in mM): 126 NaCl, 2.5 KCl, 1.2 MgCl2, 1.2 NaH2PO4, 2.4 CaCl2, 11 glucose, 25 NaHCO3. To study spontaneous glutamate-mediated excitatory postsynaptic currents (sEPSCs), the recording pipettes were filled with internal solution of the following composition (mM): K+-gluconate (125), NaCl (10), CaCl2 (1.0), MgCl2 (2.0), 1,2-bis (2-aminophenoxy) ethane-N, N,N, N-tetra acetic acid (BAPTA; 0.5), HEPES (19), GTP; (0.3), Mg-ATP; (1.0), adjusted to pH 7.3 with KOH. Bicuculline (10 µM) was added to the external solution to block GABAA-mediated transmission.

To study GABA-mediated spontaneous inhibitory postsynaptic currents (sIPSCs), the recording pipettes were filled with internal solution of the following composition (mM): 110 CsCl, 30 K + − gluconate, 1.1 EGTA, 10 HEPES, 0.1 CaCl2, 4 Mg-ATP, 0.3 Na-GTP. MK-801 and CNQX were added to the external solution to block, respectively, NMDA and non-NMDA glutamate receptors.

The detection threshold of spontaneous currents was set at twice the baseline noise. Offline analysis was performed on spontaneous synaptic events recorded during fixed time epochs (1–2 min, three to five samplings), sampled every 5–10 min. Only cells that exhibited stable frequencies in control (less than 20% changes during the control samplings) were used for analysis. For kinetic analysis, events with peak amplitude between 10 and 50 pA were grouped, aligned by half-rise time, normalized by peak amplitude, and averaged to obtain rise times and decay times.

### Real time PCR (qPCR)

Total RNA was extracted from treated BV2 cells according to the standard miRNeasy Micro kit protocol (Qiagen). The RNA quantity and purity were analyzed with the Nanodrop 1000 spectrophotometer (Thermo Scientific). The quality of RNA was assessed by visual inspection of the agarose gel electrophoresis images. Next, 900 ng of total RNA was reverse-transcribed using high-capacity cDNA reverse transcription kit (Applied Biosystem) according to the manufacturer’s instructions and 27 ng of cDNA were amplified and each reaction of amplification was performed in triplicates with SensiMix II Probe Kit in triplicate using the Applied Biosystem 7900HT Fast Real Time PCR system. The expression of IL-9R (Mm00434313_m1) and TNF (Mm00443258_m1) mRNAs were evaluated by using TaqMan technology and data were represented as 2-ΔΔCt. β-actin (Mm00607939_s1) was used as endogenous control for mRNA normalization.

### Immunofluorescence and confocal microscopy

Mice were deeply anesthetized and intracardially perfused with ice-cold 4% paraformaldehyde (PFA) at 21–25 dpi (*N* = 3 per group). Collected brains were post-fixed in 4% PFA for 2 h and equilibrated with 30% sucrose for at least one night. Thirty-micrometer-thick coronal sections were serially cut on a frozen microtome including the whole striatum to perform immunofluorescence experiments as previously described (20). Brain regions were identified using a mouse brain atlas, and for each animal, at least five serial sections were used for immunofluorescence as described above. The following primary antibodies were used overnight at 4 °C in Triton X-100 0.25%: rabbit anti-Iba1 (1:750, Wako), rabbit anti-GFAP (1:500, Dako), goat anti-DARPP-32 (1:1000, R&D systems), mouse anti-IL-9 receptor (1:200, Santa Cruz Biotechnology) and rat anti-CD3 (1:300, Biorad). After being washed, sections were incubated with the secondary antibody: Alexa-488 conjugated donkey anti-Rabbit, anti-Gt or anti-Rat (1:200, Invitrogen); Alexa-647 conjugated donkey anti-Rabbit (1:200, Invitrogen); Cy3-conjugated donkey anti-mouse (1:200, Jackson) for 2 h at RT. Nuclei were counterstained with DAPI (1 µl/ml; Sigma Aldrich). Images were acquired using a Nikon Eclipse TI2 confocal laser-scanner microscope with 20x and 40x objectives and were processed using ImageJ software. All images acquired by the confocal laser-scanner microscope had a pixel resolution of 1024 × 1024. Z-stack acquisitions (20x objective, zoom 1x with 2 μm interval for a total of 17 steps) were made applying the same intensity and exposure time. A large image function that generates a single high-magnification image (capturing 2 images) was made to detect IBA1^+^ and GFAP^+^ cells. In the z-projections, to evaluate microglia and astroglia density the number of IBA1^+^ and GFAP^+^ cells, respectively, were automatically determined and divided by the area covered by the Roi. Data were expressed as the number of cells per mm2. All images were processed using ImageJ software and were adjusted for reducing noise by applying smooth and background subtraction as required by the NIH ImageJ. ImageJ software was used to generate intensity threshold images (binary images), setting equal intensity thresholds between groups. A 40x objective (zoom 2x with 1 μm interval for a total of 30 steps) was used to detect IL-9 receptor expression in DARPP-32^+^, IBA1^+^, GFAP^+^ or CD3^+^ cells. A 40x objective zoom 1x with 2 μm interval for a total of 21 steps (with a large image function that generates a single high-magnification image capturing 4 images) was used to detect IBA1 and TNF. Binary images of IBA1 ImageJ software were used to quantify the microglial total area (IBA1^+^ surface %) in z-projected images and the measure of microglial surface was calculated by the software. Binary images on single steps of IBA1 and TNF were used to generate a colocalization mask to visualize IBA1 and TNF colocalization. Then single images were z-projected (Mk, TNF/IBA1) and merged on the binary images of IBA-1. Mk total area (TNF/IBA-1 surface %) was calculated by the software.

### IL-9 treatment

**Fig. 1:**
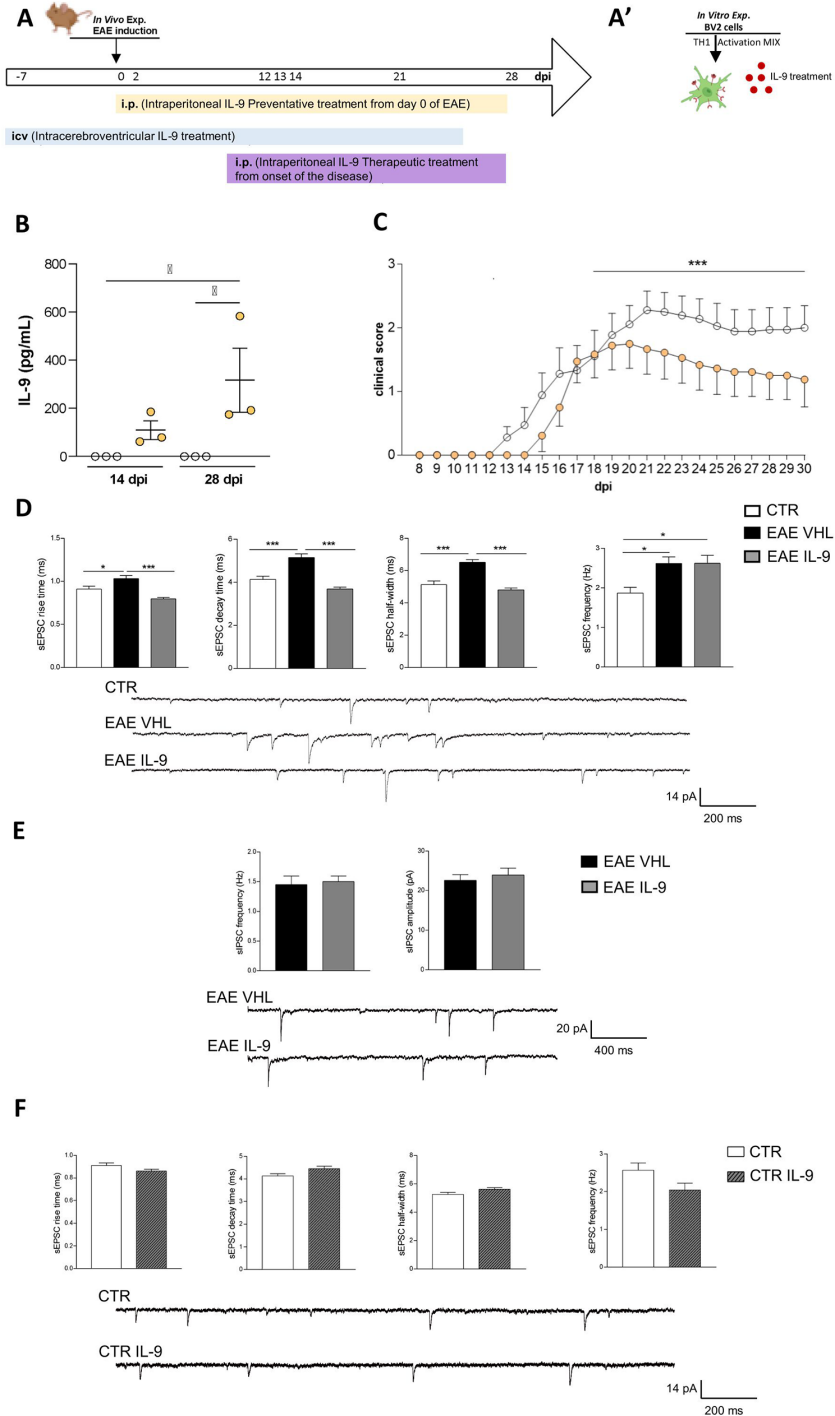
IL-9 effects on EAE disease and striatal synaptic transmission. **A-A’)** Schematic representation of the experimental design. **A)**In vivo experiments. Three in vivo treatments were performed at different times of EAE disease: peripheral intraperitoneal (i.p.) injections of IL-9 or vehicle (VHL) was carried out starting both from the day of EAE induction (preventative treatment, in yellow) and from the day of disease onset (therapeutic treatment, in purple). Central intracerebroventricular infusion of IL-9 or VHL was performed starting one week before immunization for four weeks (in blue). **A’)**In vitro experiments. BV2 immortalized murine microglial cells were activated with a Th1 Mix and treated with IL-9. **B)** The graph shows the clinical course of EAE mice treated with IL-9 (200 ng/mouse/day) or VHL (PBS) starting from the day of EAE induction (one of two different experimental sets). IL-9 treatment significantly ameliorated EAE disease progression. EAE VHL *N* = 9, EAE IL-9 *N* = 8; Mann-Whitney test from day 18 to 30 dpi, ***p < 0.001. **c)** Plasma levels of IL-9 in EAE mice following i.p. injections of IL-9 or VHL. EAE VHL *N* = 3, EAE IL-9 *N* = 3 unpaired T-test *p < 0.05. **D-F)** Electrophysiological properties recorded by whole-cell patch clamp technics from MSNs of EAE mice after ip injections from 0 dpi. Rise time, decay time and half width of glutamatergic currents, were increased in EAE striatum and were completely rescued in EAE IL-9 mice (**D**), CTR *n* = 7, EAE VHL *n* = 15, EAE IL-9 *n* = 18; one-way ANOVA followed by Tukey’s post hoc, *p < 0.05, ***p < 0.001; The frequency of the glutamatergic currents was unaffected by IL-9 treatment in EAE mice (**D**) CTR *n* = 9, EAE VHL *n* = 12, EAE IL-9 *n* = 12. Frequency and amplitude of GABAergic transmission were comparable between IL-9 and vehicle EAE-treated mice (**E**) EAE VHL *n* = 15, EAE IL-9 *n* = 9; Unpaired T-test *p* > 0.05. Ex vivo treatment of corticostriatal slices from control mice with IL-9 (100 μm, 1 h) didn’t impact on glutamatergic synaptic transmission (**F**), Frequency: CTR *n* = 14, CTR IL-9 *n* = 17; kinetic parameters: CTR *n* = 10, CTR IL-9 *n* = 24; unpaired T-test *p* > 0.05. Example of electrophysiological traces on the bottom

As schematized in experimental design (Fig. 1A-A’) three different in vivo treatments were performed at different times of EAE disease. The following intraperitoneal (ip) treatments were performed: peripheral treatment with IL-9 (200ng/mouse/day; Peproteck) and PBS as vehicle starting at 0 dpi (preventative treatment) and at onset of EAE (around 12–14 dpi, therapeutic treatment). Central treatment was performed by implanting, one week before immunization, a subcutaneous osmotic minipumps allowing continuous intracerebroventricular (icv) infusion of either IL-9 (30ng/mouse/day) or vehicle, for 4 weeks. Alzet osmotic minipumps (model 1004; Durect) connected via catheter tube to an intracranial cannula (Brain Infusion Kit 3; Alzet), delivered vehicle or IL-9 into the right lateral ventricle at a continuous rate of 0.11 µl/h. The coordinates used for icv minipump implantation were as follows: anteroposterior = − 0.4 mm from bregma; lateral = − 1 mm; depth: 2.5 mm from the skull.

For the ex vivo electrophysiological experiments, fresh striatal slices were incubated with IL-9 (100 μm), for 1 h. For BV2 experiments, 24 h activated BV2 cells were treated with IL-9 (100 μm) for 6 h.

### Statistical analysis

Statistical analysis was performed with Prism GraphPad version 9.0. Data distribution was tested for normality by using Kolmogorov–Smirnov test. Non-normally distributed data were analyzed through non-parametric tests. Differences between two groups were analysed using two-tailed Unpaired Student’s t test or Mann Whitney test, as appropriate. Multiple comparisons were performed by ANOVA followed by Tukey’s HSD. The two-way ANOVA for repeated measures was used to assess the interaction between time and treatment. Data were presented as the mean ± S.E.M, unless otherwise specified. The significance level was established at *p* < 0.05. Throughout the text “N” refers to the number of animals. For electrophysiological experiments, quantitative immunofluorescence, and biochemical analysis “n” refers to the number of slices or BV2/BV2 medium samples.

## Results

### Peripheral in vivo administration of IL-9 ameliorates EAE motor disability and striatal inflammatory synaptopathy

In order to clarify the pharmacological role of IL-9 in EAE pathology, we treated EAE mice by ip injections of IL-9 or VHL, starting from the day of EAE induction. We observed that daily systemic administration of this cytokine, which significantly increases IL-9 blood levels (Fig. 1C), ameliorated EAE disease promoting a beneficial impact on motor disability during the acute phase of the disease (Fig. 1B).

Then, we evaluated the impact of daily treatment with IL-9 on synaptic damage. Indeed, the striatum of EAE mice is characterized by an unbalance between glutamatergic and GABAergic transmission [7, 25]. Whole-cell voltage-clamp recordings from medium spiny neurons (MSNs) revealed that IL-9 completely rescued EAE-induced kinetic alterations of spontaneous postsynaptic excitatory currents (sEPSCs), in terms of rise time, decay time and half width (Fig. 1D). Conversely, the frequency of glutamatergic (Fig. 1D) and GABAergic (Fig. 1E) EAE transmissions were unaffected by the treatment.

To better characterize this synaptic effect of IL-9, we performed patch-clamp recordings in corticostriatal slices of healthy mice incubated with IL-9 or vehicle. Kinetic and frequency parameters of spontaneous glutamatergic currents were similar between IL-9-treated and untreated slices (Fig. 1F), suggesting a peculiar synaptic effect of IL-9, only in the context of neuroinflammation.

**Fig. 2:**
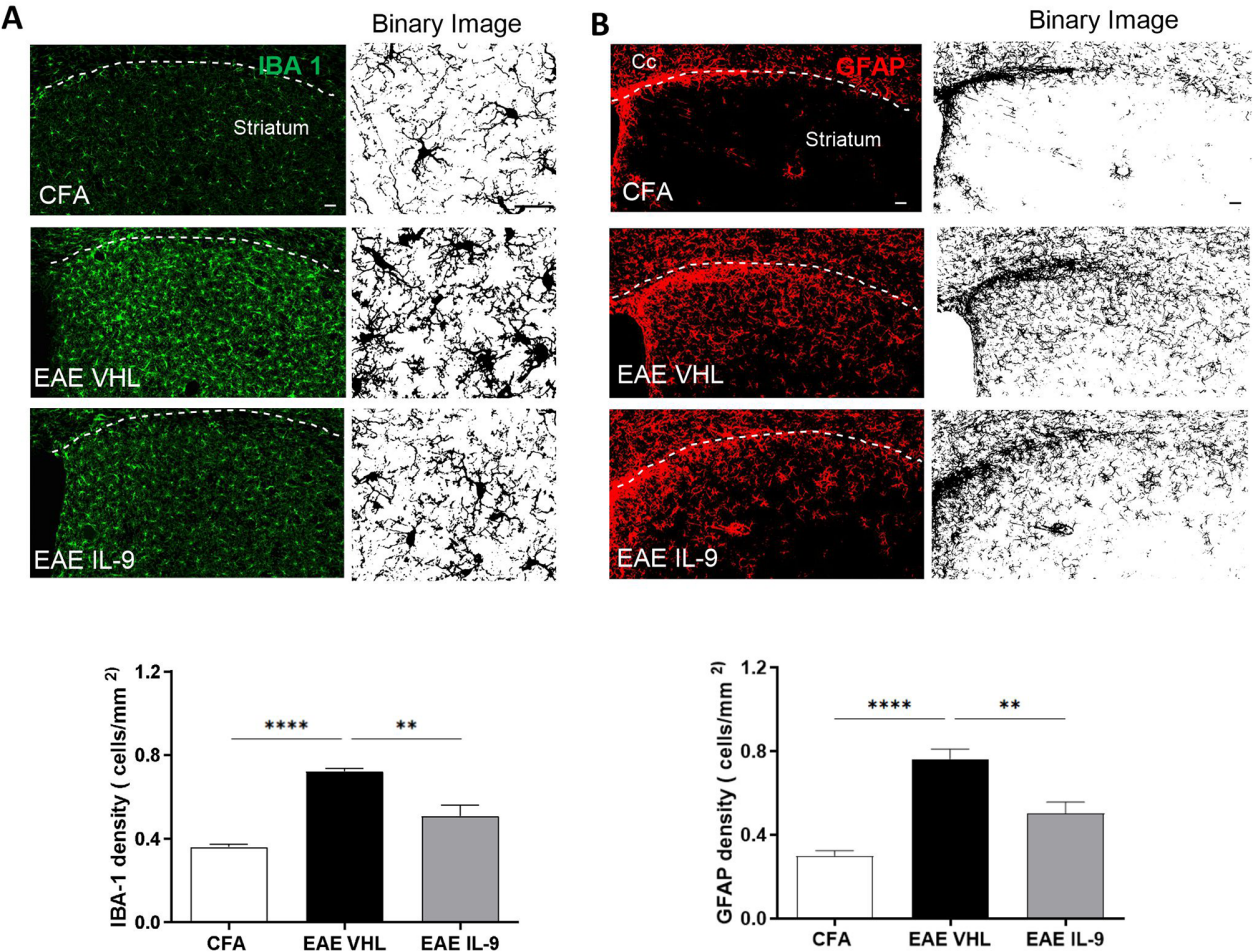
IL-9 effects on EAE striatal microgliosis and astrogliosis. **A-B)** Representative confocal microscopy images of coronal corticostriatal slices of CFA and EAE mice treated with ip injections from 0 dpi, stained with IBA1 (green fluorescence, **A**) and GFAP (red fluorescence, **B**) antibodies. Scale bar: 40 µm. Black and white binary images of IBA-1 (**A**) and GFAP (**B**) clearly highlight IL-9 effect on microgliosis and astrogliosis, respectively. **A’-B’**) Both IBA1^+^ cell density and GFAP^+^ cell density are reduced in the striatum of EAE-IL9 mice (*N* = 3, *n* = 11) compared to EAE-VHL mice (*N* = 3, *n* = 10), reaching values comparable to CFA mice (*N* = 2, *n* = 6). One-way ANOVA followed by Tukey’s post hoc, ** *p* < 0.01, **** *p* < 0.0001

Brain resident immune cells, like microglia and astrocytes, are the main mediators of EAE inflammatory synaptopathy. Thus, we performed a detailed immunofluorescence analysis of microgliosis and astrogliosis extent in the striatum of CFA and EAE mice. Quantification of microglia and astroglia density showed a significant reduction of both IBA1^+^ and GFAP^+^ cells in EAE-IL-9 mice compared to EAE-VHL mice, as highlighted in the binary images (Fig. 2).

Overall, these results reveal a beneficial effect of IL-9 ip treatment on EAE clinical disability, striatal synaptopathy and neuroinflammation.

### IL-9 mediated neuroprotection is independent of its peripheral immune effect

**Fig. 3:**
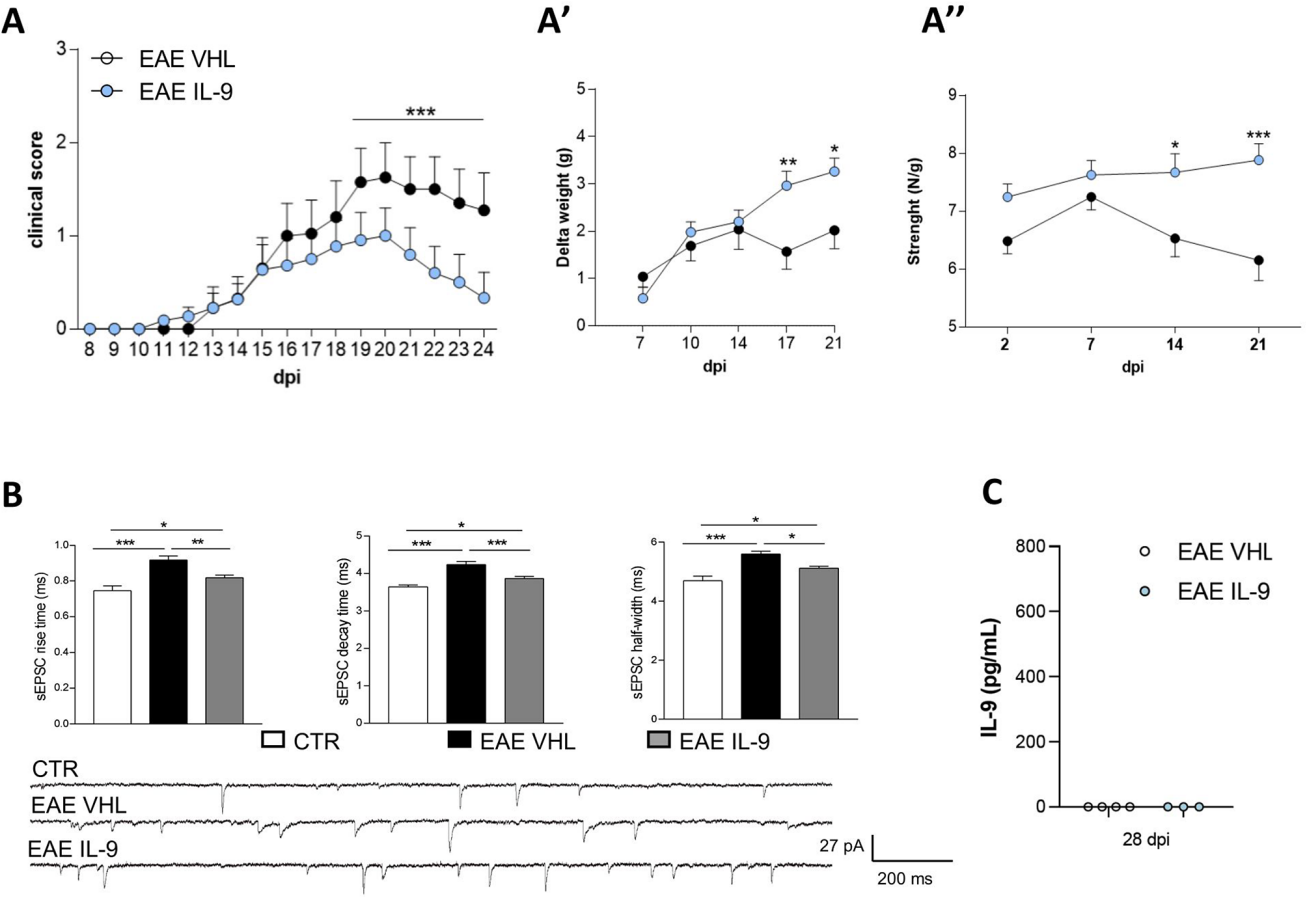
IL-9 icv treatment impact on EAE clinical disability and striatal synaptopathy. **A)** The graph shows the clinical course of EAE mice treated for four weeks with IL-9 (30ng/mouse/day) or VHL, delivered by icv infusion. IL-9 icv treatment significantly ameliorated EAE disease progression. EAE VHL N = 10, EAE IL-9 N = 11; Mann-Whitney test from 19 to 24 dpi, *** p < 0.001). **A’)** The graph shows the beneficial impact of IL-9 on body weight change obtained by comparing the body weight measured at 7,10,14,17 and 21 dpi with respect to the 0 dpi value. EAE VHL *N* = 10, EAE IL-9 *N* = 11, 2way ANOVA followed by Bonferroni’s multiple comparisons, 17 dpi **p < 0.01 and 21 dpi *p < 0.05. **A’’)** The grip strength performance was significantly ameliorated by IL-9 treatment in EAE mice after 14 and 21 dpi. EAE VHL *N* = 10, EAE IL-9 *N* = 11, 2way ANOVA followed by Bonferroni’s multiple comparisons, 14 dpi **p < 0.01 and 21 dpi ***p < 0.001. **B)** The increased kinetic properties (rise time, decay time, and half width) of EAE sEPSCs were rescued in EAE-IL9 mice. CTR *n* = 17, EAE VHL *n* = 12, EAE IL-9 *n* = 21; one-way ANOVA followed by Tukey’s post hoc, *p < 0.05, **p < 0.01, ***p < 0.001. On the bottom electrophysiological traces representative of glutamatergic currents recorded from MSNs of EAE VHL and EAE IL-9 mice. **C)** Plasma levels of IL-9 in EAE mice following icv injections of IL-9 or VHL. EAE VHL *N* = 4, EAE IL-9 *N* = 3, unpaired T-test *p* > 0.05

**Fig. 4:**
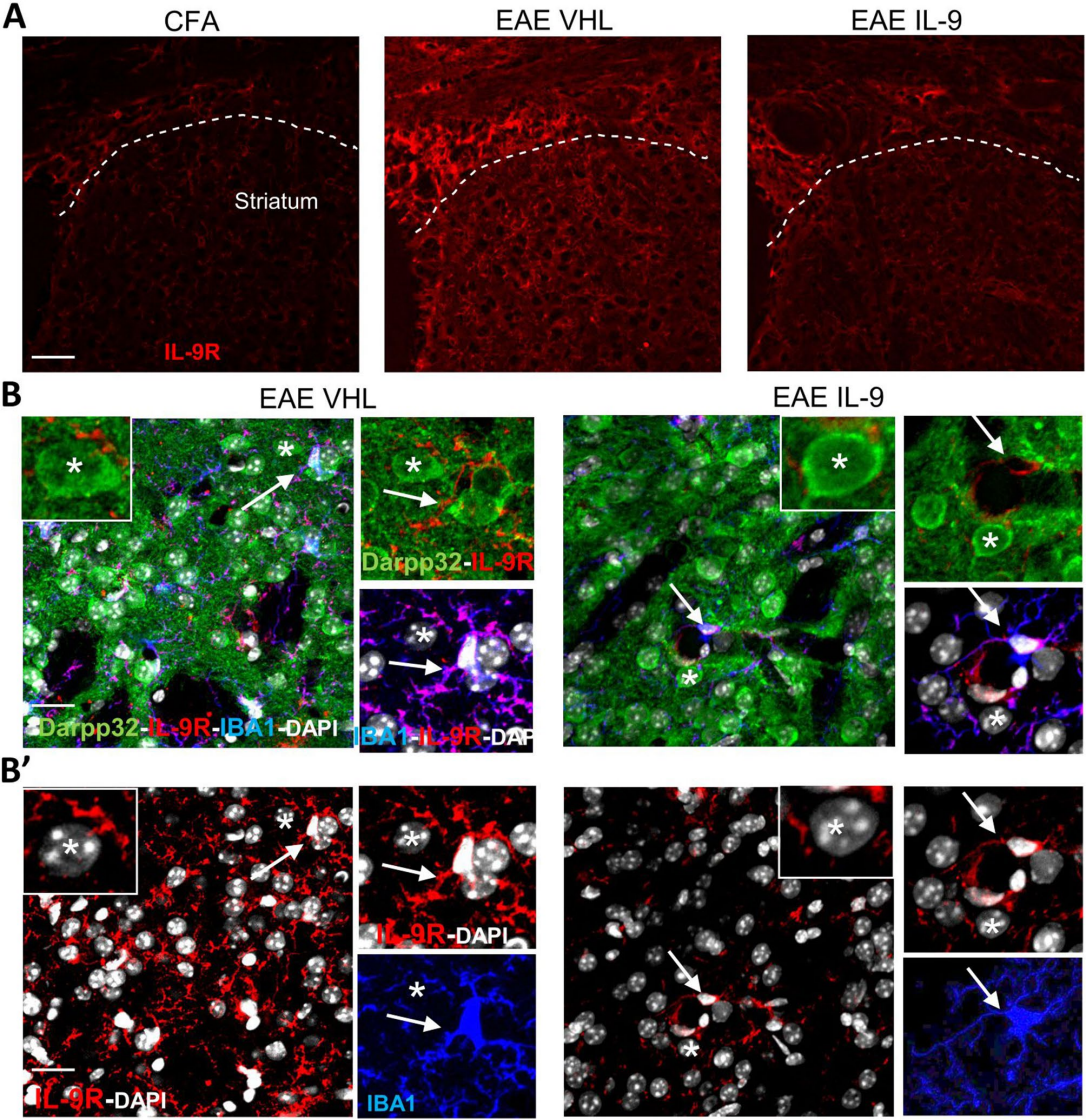
Characterization of IL-9 receptor in EAE striatum. **A)** Representative confocal microscopy images of coronal corticostriatal slices stained with IL-9R antibody. The expression of IL-9R is markedly increased in the striatum and corpus callosum of EAE mice compared to CFA condition and is reduced following IL-9 treatment. **B)** Triple immunostaining of coronal striatal sections (in gray DAPI nuclei) showing the expression of IL-9R (red fluorescence) in IBA1-positive microglia/macrophage cells (blue fluorescence) and DARPP32-positive neurons (green fluorescence) in EAE VHL and EAE IL-9 mice. As highlighted by the white asterisks IL-9R expression seems to be undetectable on neuron body cells while white arrows show the high expression of IL-9R by microglia. **B’)** Double immunostaining for IBA1 and IL-9R showing the marked reduction of IL-9R expression in IL-9 treated mice and the strong expression of IL-9R in microglia cells. Scale bar: 20 μm

The amelioration of EAE motor disability, coupled with the reductions of striatal neuroinflammation and improvements in glutamatergic transmission defects, highlights the beneficial influence of IL-9 within the CNS. To rule out a secondary cytokine effect into the brain promoted by the involvement of peripheral compartment, we treated for 4 weeks EAE mice with icv injections of IL-9 or vehicle. We observed that daily central treatment with this cytokine ameliorated the clinical score of the disease (Fig. 3A), resembling the effect obtained after ip treatment. Data on clinical score was corroborated by other typical clinical parameters of the EAE condition, such as body weight and neuromuscular strength. Specifically, both body weight change (Fig. 3A’) and 4 limbs strength (Fig. 3A’’) were significantly ameliorated by IL-9 treatment. Moreover, icv infusion with IL-9 replicated the anti-synaptotoxic effect observed following systemic treatment. Indeed, whole-cell patch clamp recordings showed that the increase of glutamatergic kinetics induced by EAE was rescued in EAE-IL9 (icv) mice, while sEPSC frequency was unaffected (data not shown). Importantly, IL-9 blood levels were comparable between groups (Fig. 3C), indicating a negligible IL-9 systemic involvement.

Overall, these results suggest that IL-9 ameliorates EAE clinical disability and neuronal toxicity independently of its peripheral immune effect.

### Characterization of IL-9 receptor in EAE striatum

Based on the central effect promoted by IL-9 administration, we investigated the expression of IL-9 receptor (IL-9R) in the striatum of EAE mice. Firstly, by performing immunostaining for IL-9R, we observed an overexpression of this receptor in EAE compared to CFA mice (Fig. 4A), in line with previous data obtained in EAE brain and spinal cord [15]. Moreover, we observed that IL-9 treatment reduced IL-9R staining in EAE mice (Fig. 4A and B’), and images (Fig. 4B-B’) confirmed the reduction of microgliosis following IL-9 treatment. Then, immunostaining for IL-9R combined with neuronal DARPP-32, microglia IBA-1, astroglia GFAP and infiltrating lymphocytes CD3 markers showed that microglia clearly express IL-9R (Fig. 4B-B’). Conversely, this receptor was undetectable in GFAP + cells and CD3 infiltrating lymphocytes (Supplementary Fig. 1), as well as into the cell body of medium spiny neurons (Fig. 4B), although its expression on dendrites and axons could not be excluded. These results indicate that activated microglia represent the main cellular population expressing IL-9R in the EAE striatum and that in vivo treatment with the cytokine decreases the EAE-mediated enhancement of IL-9R expression.

### IL-9 counteracts TNF mediated excitotoxicity

Based on the evidence that TNF is a critical player in the exacerbation of striatal glutamate transmission induced by EAE [7], we explored the link between IL-9 and TNF to understand if the recovery of synaptic defects mediated by IL-9 was correlated to the reduction of TNF expression in EAE brain.

**Fig. 5:**
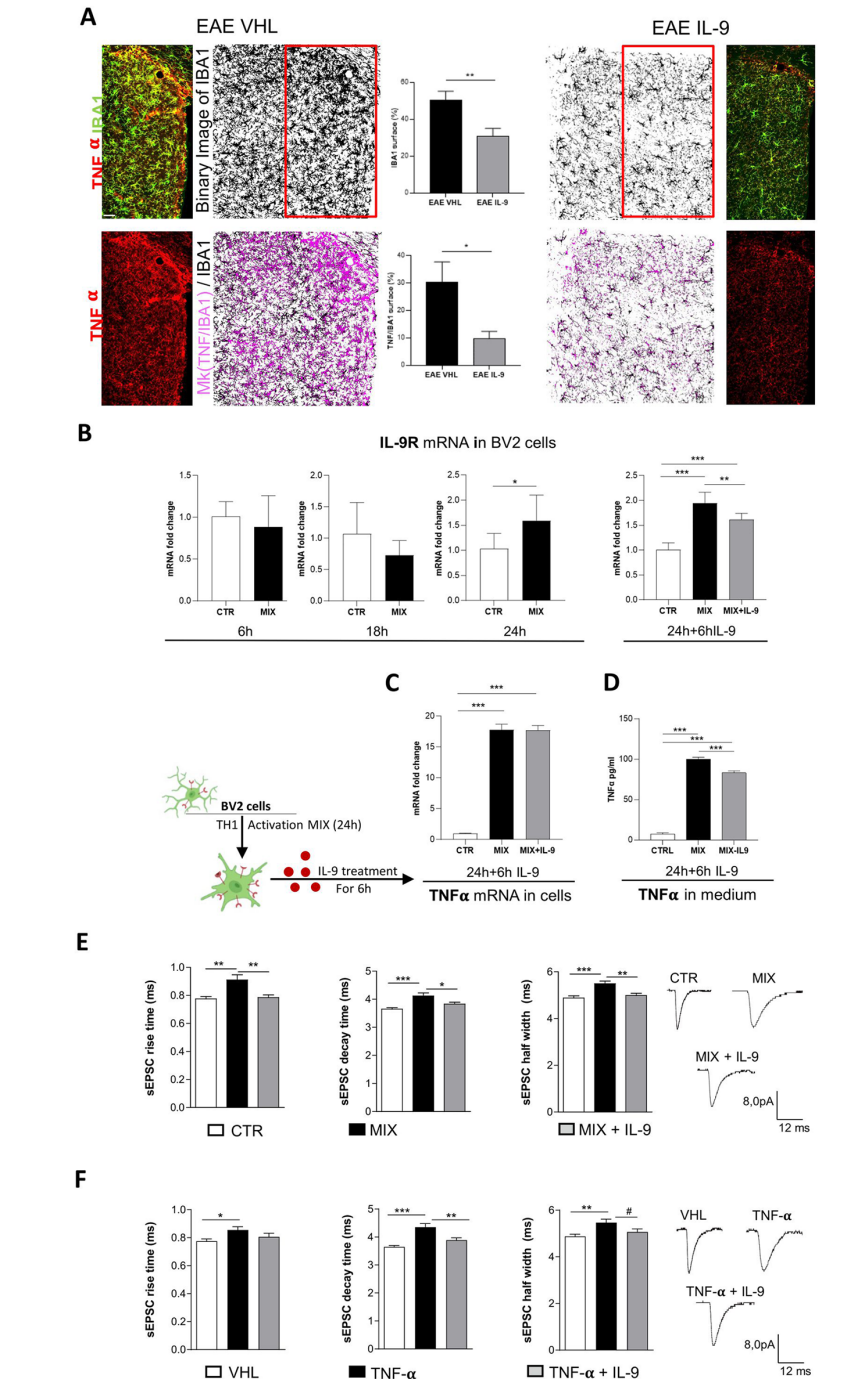
IL-9 prevents TNF detrimental effects on striatal synaptic transmission. **(A)** Representative confocal microscopy images of coronal striatal slices stained with IBA1 (green fluorescence) and TNF-ɑ (red fluorescence) showing that IL-9 treatment decreased microgliosis and TNF-ɑ expression in the striatum of EAE mice. Percentage of the area occupied by microglia is reduced in the striatum of EAE IL-9 mice compared to EAE VHL mice. The overlapping area, mask (mk TNF/IBA1, magenta), is clearly reduced in microglia from EAE IL-9 striatum compared to EAE VHL group, indicating less production of TNF-ɑ from these cells. EAE VHL *N* = 3,*n* = 9, EAE IL-9 *N* = 3,*n* = 11, Unpaired two-tailed t-test, * *p* < 0.05,** *p* < 0.01. Scale bar: 20 μm. **(B)** qPCR experiments performed on BV2 microglial cells activated by Th1 Mix for 6 h, 18 h and 24 h show an overexpression of IL-9R only following 24 h of cytokine activation. *n* = 6 per condition, Unpaired t-test, * *p* < 0.05. Incubation of 24 h activated BV2 cells with IL-9 (6 h, 1 μm) significantly reduced IL-9R mRNA level. *n* = 6 per condition, one-way ANOVA followed by Tukey’s post hoc CTR *** *p* < 0.001, ** *p* < 0.01. All data are expressed as mean ± SEM and as fold change of untreated controls. **(C)** qPCR quantification of TNF-ɑ transcriptional levels reveals that IL-9 treatment of BV2 activated cells do not modulate TNF mRNA. *n* = 6 per condition, one-way ANOVA Tukey’s post hoc analysis ***p < 0.001. **(D)** TNF concentration (pg/ml) in the medium of BV2 cells non-activated, activated and activated in the presence of IL-9. Incubation with IL-9 caused the reduction of TNF protein release by activated microglia. CTRL *n* = 4, MIX (activated BV2 cells) *n* = 5, MIX + IL-9 (activated BV2 cells + IL-9) *n* = 5; one-way ANOVA Tukey’s post hoc analysis *** *p* < 0.001. **E)**In vitro activated microglia stimulated with IL-9 (MIX + IL-9) significantly ameliorated the altered rise time, half-width and decay time of sEPSC of striatal slices. Control slices (CTRL) *n* = 17, activated BV2 (MIX) *n* = 18, activated BV2 + IL-9 (MIX + IL-9) *n* = 18; unpaired T-test *p < 0.05, **p < 0.01. **F)** The increased kinetic parameters induced by TNF in striatal slices were rescued by concomitant incubation of IL-9. VHL *n* = 17, TNF + VHL *n* = 19, TNF + IL-9 *n* = 17; One-way ANOVA * *p* < 0.05, **p < 0.01, ***p < 0.001; Unpaired T-test #*p* < 0.05

Microglia cells are the main source of TNF in the inflamed EAE brain [26], and selectively express IL-9R (present study). Thus, we performed co-immunolabeling of the microglia marker IBA1 and of TNF on EAE striatal slices. As expected, we observed a strong expression of TNF by IBA-1^+^ cells and the analysis of the total area covered by microglia cells confirmed the impact of IL-9 on microglia density reduction (Fig. 5A). IL-9 treatment significantly reduced TNF expression from microglia, as highlighted by colocalization mask (mk-magenta) analysis (Fig. 5A).

Next, to better define the implication of microglia cells on IL-9-mediated neuroprotective effects, we investigated the impact of IL-9 on BV2 immortalized murine microglial cells. Indeed, BV2 stimulation with a proinflammatory Th1 cytokine mix (IL-1β, IFNγ, and TNFα) well resembles EAE cytokine microenvironment and promotes inflammatory synaptopathy typical of EAE [7, 27]. We stimulated BV2 cells for 6 h, 18 h and 24 h. qPCR analysis revealed a significant increase of IL-9R mRNA levels at 24 h in BV2 treated cells compared to control conditions (Fig. 5B), suggesting the suitability of BV2 experimental model to study IL-9 effects. Therefore, following 24 h of activation, BV2 cells were treated with IL-9 for 6 h. IL-9R mRNA levels in activated BV2 cells were strongly reduced by IL-9 treatment, resembling the effect observed in EAE condition (Fig. 5B). Then, we evaluated the impact of IL-9 on TNF release by measuring TNF protein level in the conditioned medium of BV2 cells. As indicated by the immunofluorescence analysis performed in EAE striatal slices, ELISA immunoassay confirmed that IL-9 treatment significantly reduced BV2 TNF release induced by Th1 cytokines mix (Fig. 5D). Conversely, TNF mRNA levels were similar between IL-9 and vehicle BV2-trated cells (Fig. 5C), suggesting a post-transcriptional effect.

As previously demonstrated, activated BV2 cells promote the increase of sEPSC duration (inducing the same alterations seen in EAE), through a mechanism dependent on TNF release. Thus, we recorded corticostriatal slices incubated with activated BV2 cells treated with IL-9 or vehicle. IL-9 prevented neuronal toxicity induced by activated BV2 cells, rescuing both decay time and half width parameters (Fig. 5E), demonstrating the direct impact of IL-9 on microglia-induced synaptic transmission.

### The dual role of IL-9 in the modulation of TNF mediated excitotoxicity

To better characterize the biological interconnection between IL-9 and TNF, the downstream impact of IL-9 on TNF synaptic toxicity was also investigated. To this aim, striatal slices from healthy mice were incubated with TNF in the presence of IL-9 or vehicle. Electrophysiological alterations induced by exogenous TNF in control striatal slices were rescued by concomitant incubation with IL-9 (Fig. 5F). This effect was particularly evident in terms of decay time, as slices treated with both TNF and IL-9 reached values similar to the control condition (vehicle) and significantly lower compared to the TNF condition. Although less pronounced, a similar effect was also observed for the half-width kinetic parameter (Fig. 5F).These results suggest a neuronal role for IL-9, unveiling a functional interplay between TNF and IL-9 signaling.

In summary, our findings indicate that IL-9 contrasts TNF-dependent synaptotoxicity via multiple actions: influencing microglial activation, reducing TNF expression and release, and interfering with the neurotoxic effects of TNF.

### The impact of IL-9 treatment on TREM2 expression

Clinical and preclinical data pointed out triggering receptor expressed on myeloid cells-2 (TREM2) as a signature of alternative microglia/macrophage M2 activated profile that promotes the resolution of inflammation and clearance of myelin debris in MS disease [28–30].

**Fig. 6:**
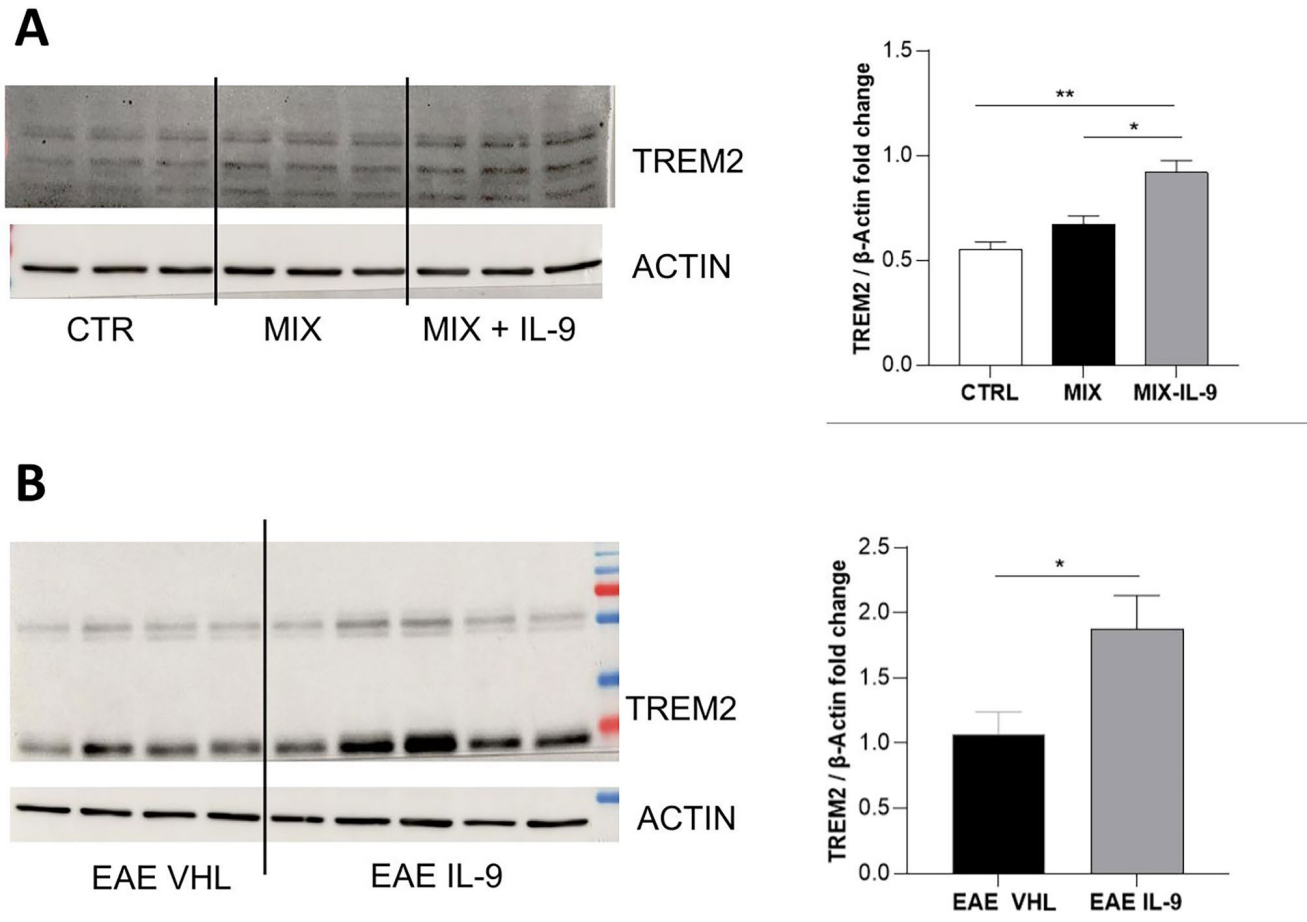
IL-9 modulates TREM2 expression in BV2 and in EAE slices. Western blot comparing the probe for anti-TREM2 antibody in BV2 cells (CTRL, 24 h of MIX activation and 24 h of MIX activation + 6 h of IL-9 treatment) and in EAE VHL and EAE-IL9 striatal extracts (i.p. treatment from day 0). Densitometric analysis of the bands shows an increased TREM2 content, revealing that IL-9 induces an upregulation of TREM2 level in both BV2 cells (A) and EAE striatum (B). One-way ANOVA and Unpaired T-test; * *p* < 0.05, **p < 0.01. *n* = 3 per condition for in vitro experiment and *N* = 4–5 per group in EAE mice

To investigate the impact of IL-9 on the microglia activated profile, we evaluated TREM2 levels in both ex vivo and in vivo conditions. Specifically, Western blot experiments were performed on BV2 cells activated as previously described (24 h of activation) and treated with IL-9 or VHL (6 h). The analysis revealed that IL-9 treatment significantly increased TREM2 expression compared to control and stimulated conditions (Fig. 6A). A similar result was obtained also in EAE mice. Indeed, TREM2 expression in the striatum of EAE mice systematically treated with IL-9 and sacrificed at 24 dpi was significantly higher compared to EAE-VHL animals (Fig. 6B). Notably, TREM2 was shown to inhibit inflammatory response in microglia cells by reducing the production of inflammatory mediators such as TNF [31, 32]. Altogether these findings support the beneficial role of IL-9 on microglia-mediated toxicity in MS disease, suggesting a mechanism dependent on modulation of TREM2 expression.

### Effectiveness of IL-9 therapeutic treatment in EAE

**Fig. 7:**
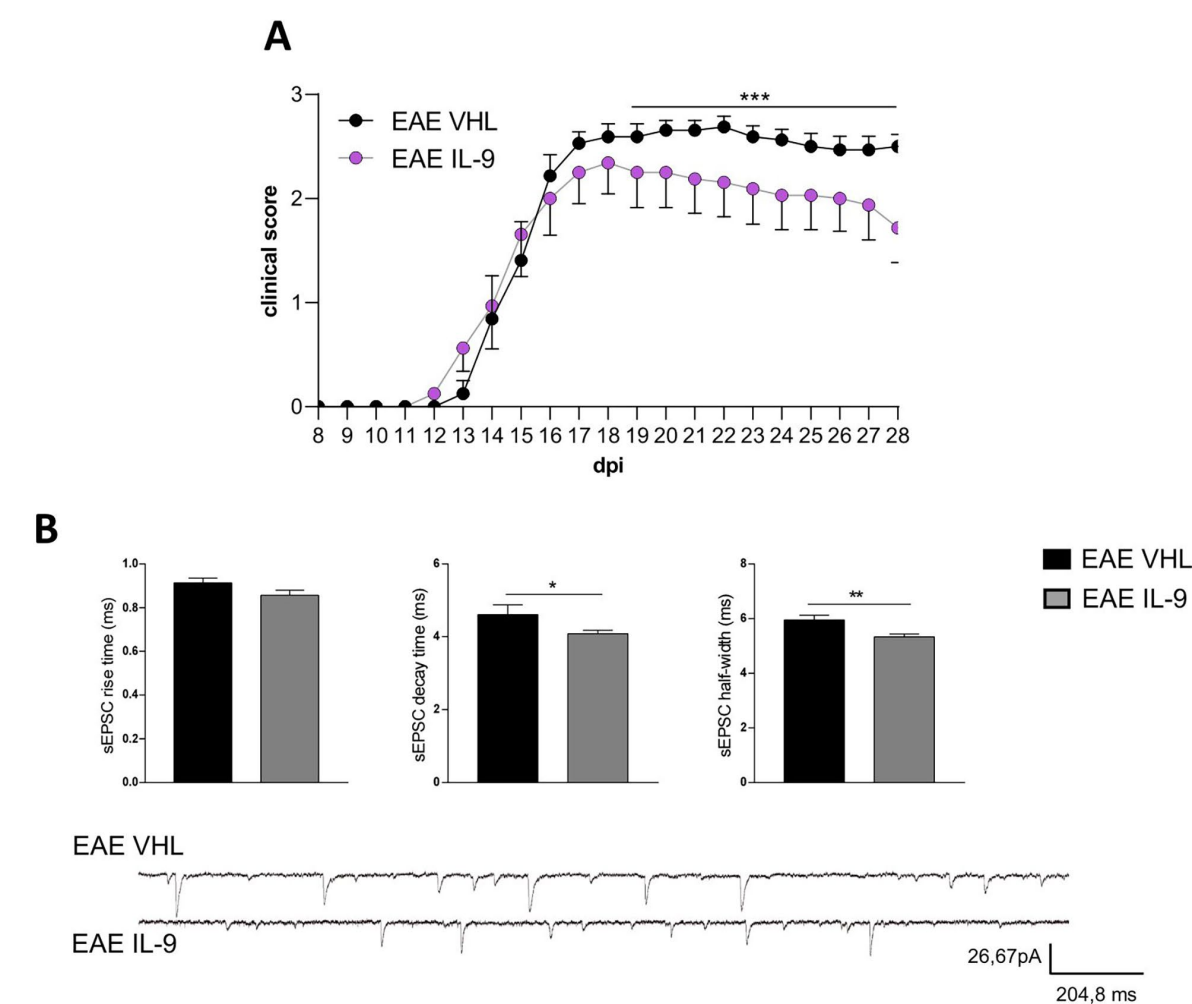
Therapeutic treatment with IL-9 ameliorates EAE disease. **(A)** The graph shows representative clinical courses of EAE IL-9 and EAE VHL mice treated starting from the day of disease onset. IL-9 therapeutic treatment significantly ameliorated EAE disease progression. EAE VHL *N* = 8, EAE IL-9 *N* = 8; Mann-Whitney test from 19 to 28 dpi, *** *p* < 0.001). **(B)** MSNs from mice therapeutically treated with IL-9 (grey) showed reduced half width and decay time compared to EAE VHL (black) condition. EAE VHL *n* = 7, EAE IL-9 *n* = 18; Unpaired T-test *p < 0.05. **p < 0.01

To investigate the translational relevance of IL-9 treatment in MS disease, we performed a therapeutic treatment using daily ip injections of IL-9 in EAE mice, starting from the onset of clinical disease. Similar to the results observed in other experimental conditions, this therapeutic approach significantly ameliorated EAE clinical disability as well (Fig. 7A). In parallel, IL-9 was effective in mitigating EAE-related striatal synaptopathy, leading to a significant reduction in the decay time and half-width of sEPSC (Fig. 7B). This experiment unequivocally demonstrates the beneficial effects of IL-9 in EAE pathology and underscores its therapeutic potential in the disease.

## Discussion

In the present study, we investigated the role of IL-9 in the mouse model of MS, uncovering for the first time a relevant neuroprotective role of this cytokine. The beneficial effect of IL-9 in MS disease was previously suggested in a clinical study where CSF levels of IL-9 have been negatively correlated with neurodegeneration, inflammatory activity and disease severity in RRMS patients [12], while information on progressive patients is missing. Most of the preclinical experiments performed to understand the IL-9 role in MS disease focused on its peripheral immunomodulatory effect and reached controversial results. Genetic deletion of IL-9R in EAE mice was shown to both increase [17, 18] and attenuate [21] the severity of EAE. The reduction of physiological IL-9 levels by anti-IL-9 monoclonal antibody (ɑIL-9) reduced EAE severity, in both active [19, 20] and passive [20] EAE. Noteworthy, the anti-IL9 treatment promoted a feedback enhancement of IL-9 production from T cell [19]. Furthermore, stimulation of IL-9 production from T cells ameliorated or worsened EAE clinical course in mice treated before or concomitantly with EAE induction, respectively [21]. To clarify the role of IL-9 in EAE disease and to discriminate peripheral and central functions of this cytokine, we performed different in vivo pharmacological treatments, combined with ex vivo and in vitro data.

Besides demyelination, the complex alterations of excitatory and inhibitory transmission induced by neuroinflammation is increasingly considered as a relevant pathogenic and neurodegenerative mechanism in EAE and MS brains [3]. Synaptic damage occurs in the absence of overt demyelinating lesions, and even before the appearance of the EAE-associated neurological deficits. Among proinflammatory cytokines, TNF plays a crucial role in this inflammatory synaptopathy, mediating the increase of glutamatergic postsynaptic duration in the striatum of EAE mice [7]. Microglia cells are the main source of inflammatory mediators in the EAE/MS brains and are convincingly recognized to exert a crucial role in the pathogenic mechanisms leading to CNS inflammation and damage in MS [7, 33]. Activated microglia were found in brain lesions of MS patients, in all stages of the disease, and were thought to contribute in recruitment of adaptive immune cells to the CNS [34]. Diffuse microglial and astroglial reactions indicate that the innate immune system is chronically activated in the gray matter and features of degeneration-associated/pro-inflammatory states of microglia increase with chronicity of the disease [35]. Importantly, persistent inflammation triggers uncontrolled synaptic activation and excitotoxicity [36]. We have previously demonstrated that activated microglia incubated on brain slices of healthy mice mimicked the aberrant glutamatergic transmission observed in the brain of EAE mice [7, 37–39]. Specifically, TNF released by reactive microglia led to enhanced AMPAR localization at the postsynaptic surface causing an increase of glutamatergic transmission [8].

Current disease modifying therapies (DMTs) exerts a limited impact on microglia cells through indirect mechanisms [40]. Understanding how to restore microglia to homeostasis and shift pro-inflammatory microglia toward an anti-inflammatory phenotype has significant implications for MS therapies, leading to new approaches to counter microglia-driven neurodegeneration in MS [35, 41]. In the present study, we demonstrate that IL-9 was able to mitigate EAE clinical disability and to reduce neuroinflammation and synaptic damage by a preferential interaction with microglial cells and TNF release and synaptic effects.

The CNS role of IL-9 is supported by the identification of IL-9 receptor in the brain. Astrocytes, microglia, oligodendrocytes and neurons were shown to express IL-9R and to be modulated after IL-9 in vitro stimulation [15, 16, 22, 42]. In this study we examined for the first time the expression of IL-9R in EAE striatum. By immunofluorescence analysis we observed that microglia/macrophage cells represent the main cellular population expressing IL-9R indicating that microglia may strongly respond to IL-9 pharmacological treatment during EAE disease. In line with this, studies on *post-mortem* brain tissues showed that IL-9R is expressed by macrophages/microglia in the CNS of MS patients [13]. Moreover, among human blood cells, infiltrating monocytes expressed the highest level of this receptor [13].

In vitro experiments showed that IL-9 reduced inflammatory properties and inhibited TNF release in LPS stimulated- human monocytes/macrophages [13, 14]. Quantitative immunofluorescence analysis performed in this study clearly demonstrates that, in the striatum of EAE mice, TNF production in microglia cells was reduced by IL-9 treatment. Experiments on immortalized murine microglial cell line BV-2 confirmed the direct interplay between IL-9 and microglia cells. Indeed, IL-9 in vitro treatment promoted both the reduction of TNF release and the rescue of synaptic modulation induced by activated BV2 cells.

In multiple sclerosis (MS), the resolution of inflammation is facilitated by the alternative M2-activated microglia/macrophage profile [28–30]. TREM2 is a reliable marker of the M2 microglia profile and plays a pivotal role in limiting central nervous system (CNS) damage. Notably, a recent clinical study uncovered a significant positive correlation between IL-9 and TREM2 levels in the cerebrospinal fluid (CSF) of relapsing-remitting MS patients [43]. Within the CNS, the TREM2 receptors appear to be exclusively expressed by microglial [44]. In EAE mice, TREM2 was shown to play an important role in limiting the extent of CNS damage, since its blockade exacerbates EAE disease.

Moreover, TREM2 expression is up-regulated during EAE, and its blockade has been shown to exacerbate the severity of the disease [29]. Furthermore, intravenous administration of TREM2-transduced myeloid precursors in EAE mice led to an improvement in clinical symptoms, reduced axonal damage, enhanced removal of degenerated myelin, and decreased gene transcription of proinflammatory cytokines, including TNF [30]. The promotion of remyelination and myelin debris clearance through TREM-2 activation on microglia was also observed in a different mouse model of MS, the cuprizone model [28].

Finally, to investigate a putative interplay between IL-9 and TNF synaptic function and not only TNF release, we explored the anti-synaptopathic activity of IL-9 in healthy corticostriatal slices incubated with TNF. Such an ex-vivo experiment showed that IL-9 blocked TNF synaptic toxicity, suggesting a further mechanism independent of TNF release and probably due to a neuronal action. Indeed, although we did not detect IL-9R signal in MSN soma, we cannot rule out an expression of this receptor in neuronal terminations.

The evidence that IL-9 significantly attenuated excitotoxicity ascribed to TNF mediated synaptic damage [7, 45, 46] leads us to propose that the beneficial effect of IL-9 observed in the EAE striatum can be, at least in part, related to its anti-TNF action. In vitro experiments on BV2 cells and ex vivo investigation on brain slices reveal two mechanisms by which IL-9 can promote this protective role: first, IL-9 reduces the release of TNF by microglia cells; second, IL-9 directly blocks the neuronal TNF effect. This synergistic effect of IL-9 on TNF mediated excitotoxicity could explain the efficacy of in vivo treatment.

The increase of IL-9R expression in EAE brain [15] and present study], indicates the high susceptibility of CNS to IL-9 pharmacological treatment during the disease. Moreover, BV2 experiments confirm the link between IL-9R expression and inflammatory microenvironment, since only a sustained inflammatory stimulus (at least 24 h) allows the increase of IL-9R. Of note, an important negative regulatory pathway has been described between IL-9 and its receptors. Indeed, IL-9 stimulation promotes the down-regulation of cell surface receptor [47]. Such negative feedback could explain our experimental data on both striatal slices and BV2 cells showing a strong reduction of IL-9R levels following its stimulation.

The clinical relevance of IL-9 treatment was finally investigated by assessing its impact when administered starting from the onset of clinical manifestation. In line with the other in vivo treatment, the cytokine significantly ameliorated the EAE disease, highlighting its therapeutic potential in MS pathology.

## Conclusions

This study reveals a central action of IL-9 in ameliorating EAE disease. Our data indicate that IL-9 attenuates neuroinflammation and synaptic damage, counteracting TNF-mediated neuronal toxicity.

The recognition that not only myelin but also synapses are early and privileged sites of damage in MS and involved in tissue loss could have profound therapeutic implications; mitigation of inflammatory synaptopathy can cause in parallel the correction of the synaptic alterations and the attenuation of motor deficits of EAE mice.

Altogether the data strengthen the relevance of an immunomodulatory and neuroprotective action of IL-9 in MS, and shed light on IL-9 modulation as an effective disease modifying therapeutic strategy to counteract neuronal and synaptic loss, as well as irreversible disability in MS.

### Electronic supplementary material

Below is the link to the electronic supplementary material.

Supplementary Material 1

Supplementary Material 2

Supplementary Material 3

## Data Availability

All the research data are available from the corresponding author on request.

## Abbreviations

ACSF: Artificial CSF
BBB: Blood-brain barrier
CNS: Central nervous system
CFA: Complete Freund’s Adjuvant
dpi: Day post-immunization
EAE: Experimental autoimmune encephalomyelitis
IL: Interleukin MSNs: medium spiny projection neurons
MS: Multiple sclerosis
MOG35-55: Myelin oligodendrocyte glycoprotein peptide 35–55
PFA: Paraformaldehyde
PGs: Prostaglandins
sEPSC: Spontaneous postsynaptic glutamatergic current
sIPSC: Spontaneous postsynaptic inhibitory current
VHL: Vehicle
TREM2: Triggering receptor expressed on myeloid cells-2
