# Supplementary figures and images for: Interleukin-9 protects from microglia- and TNF-mediated synaptotoxicity in experimental multiple sclerosis
Source: J Neuroinflammation. 2024 May 14;21:128. doi: 10.1186/s12974-024-03120-9 (PMC11092167; doi:10.1186/s12974-024-03120-9)

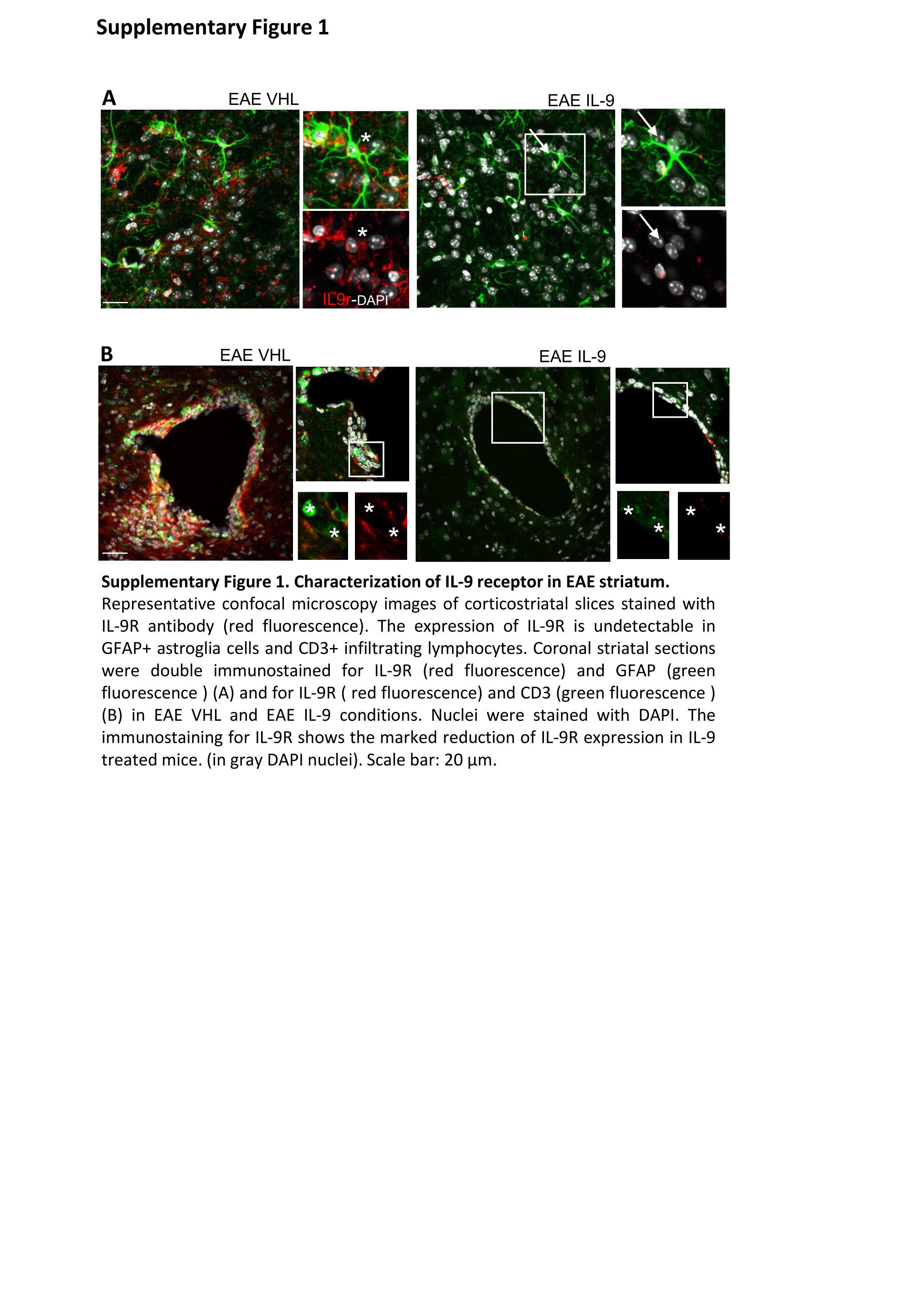

Supplement: Supplementary file 1 — Supplementary Material 1 [file 12974_2024_3120_MOESM1_ESM.jpg]

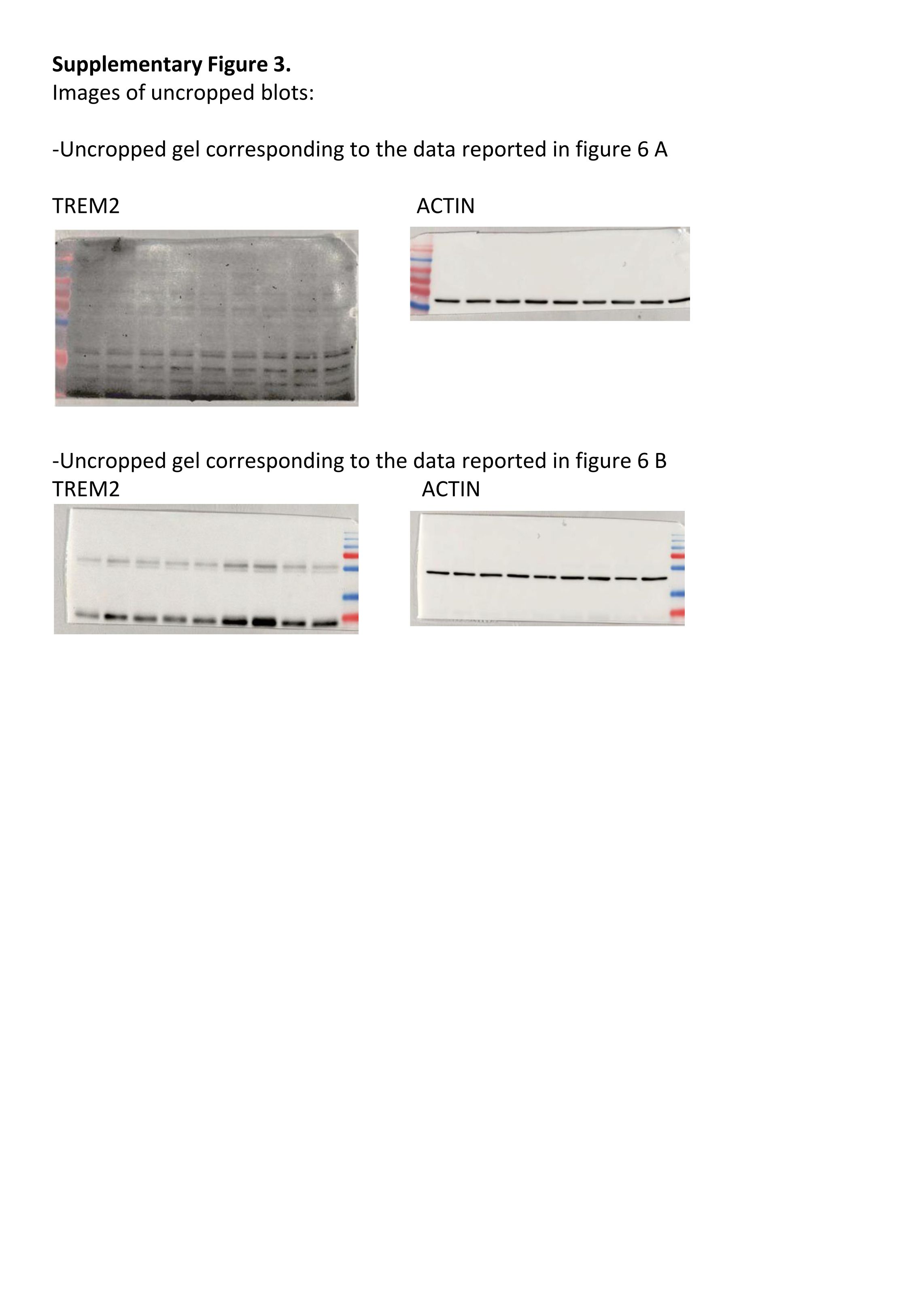

Supplement: Supplementary file 2 — Supplementary Material 2 [file 12974_2024_3120_MOESM2_ESM.jpg]

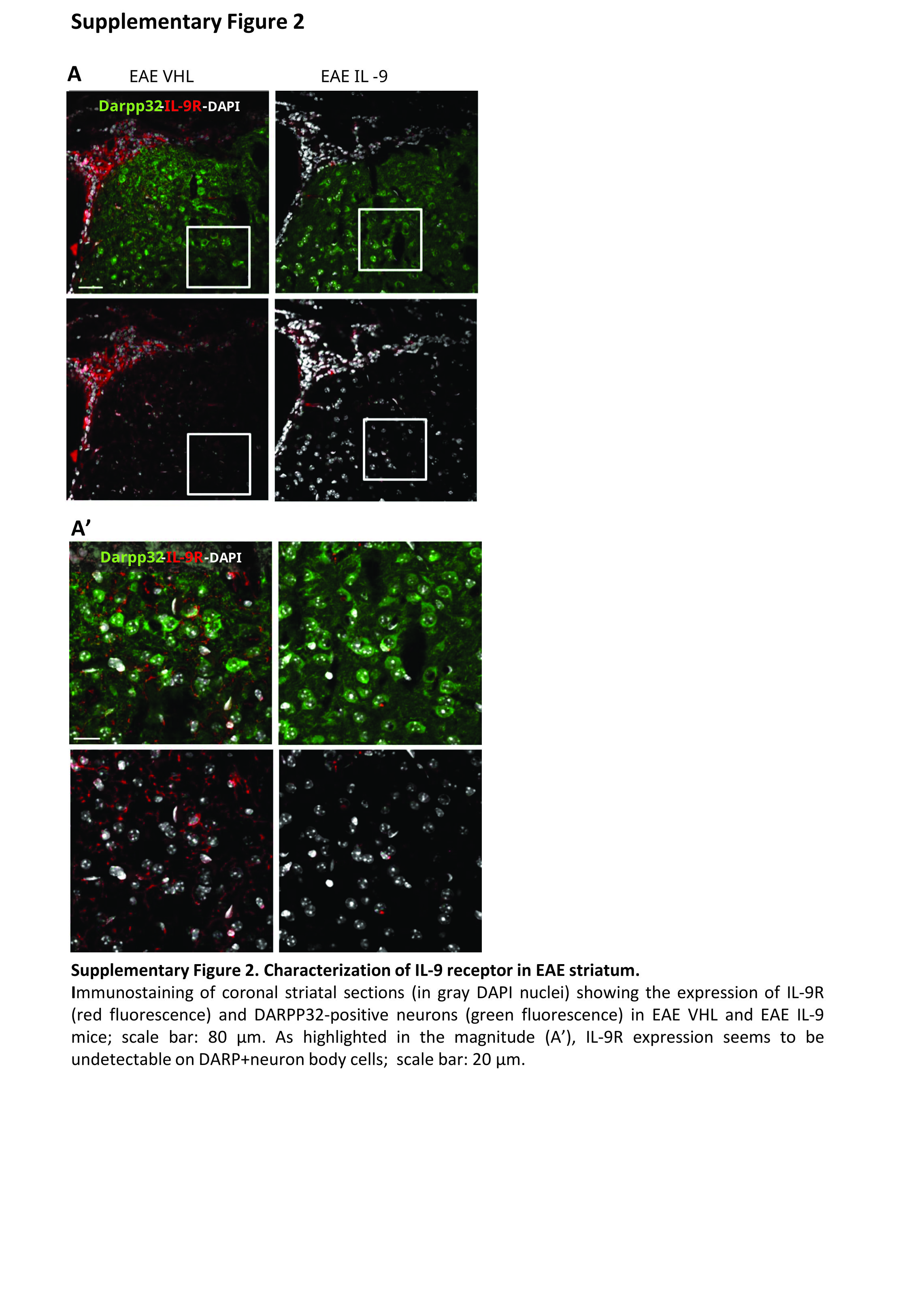

Supplement: Supplementary file 3 — Supplementary Material 3 [file 12974_2024_3120_MOESM3_ESM.jpg]
